# Nitric Oxide Enhances Drought Tolerance in *Gossypium hirsutum* L. via S-Nitrosylation of the Plasma Membrane H^+^-ATPase Isoform GhHA2 and Antioxidant Defense Activation

**DOI:** 10.3390/plants15101463

**Published:** 2026-05-11

**Authors:** Yiping Sui, Shuying Li, Xiaoli Tian, Fangjun Li, Zhaohu Li

**Affiliations:** State Key Laboratory of Plant Environmental Resilience, Engineering Research Center of Plant Growth Regulator, Ministry of Education & College of Agronomy and Biotechnology, China Agricultural University, No.2 Yuanmingyuan Xilu, Haidian District, Beijing 100193, China

**Keywords:** nitric oxide, antioxidant defense, stomatal closure, coordinated regulation, drought, cotton plant

## Abstract

(1) Background: Nitric oxide (NO) serves as a crucial signaling molecule in plant abiotic stress responses. Although its role in enhancing drought resistance in cotton has been recognized, the specific mechanisms underlying this physiological and molecular regulation remain largely unexplored. This study aims to elucidate the multi-layered mechanisms by which NO modulates drought resistance in cotton; (2) Methods: Cotton seedlings were subjected to drought stress with the application of the NO donor sodium nitroprusside (SNP). A combination of confocal laser scanning microscopy, transcriptional expression analysis, biochemical assay of enzyme activity, virus-induced gene silencing (VIGS), and in vitro protein modification assays was applied to characterize the effects of NO on the drought stress response in cotton; (3) Results: Exogenous NO significantly reinforced drought resistance in cotton seedlings by improving leaf water retention capacity and photosynthetic efficiency, eliminating excessive drought-induced reactive oxygen species (ROS), upregulating the transcription and enzymatic activity of antioxidant enzymes, and promoting stomatal closure. Mechanistically, NO triggered S-nitrosylation of the plasma membrane H^+^-ATPase isoform GhHA2, thereby enhancing its protein stability; (4) Conclusions: These findings reveal that exogenous NO orchestrates cotton drought tolerance via multiple interconnected physiological and molecular pathways, in which the activation of the antioxidant defense system and the modulation of stomatal closure serve as central regulatory mechanisms.

## 1. Introduction

Abiotic stress is a major constraint limiting agricultural productivity, with its adverse effects exacerbated by climate change [1,2]. Drought stress induces cellular water deficit in plants. Upon perceiving water deficit, plants initiate signal transduction cascades to trigger physiological and biochemical adaptations, including enhanced root water uptake, stomatal closure to reduce transpiration, and osmotic adjustment [3,4,5]. Concurrently, hormone signaling pathways (e.g., abscisic acid, ABA) and antioxidant defense systems are activated [6,7].

As a pivotal gaseous signaling molecule, NO-mediated signal transduction plays a critical role in plant responses to various abiotic stresses. Upon exposure to drought, high salinity, or extreme temperatures, plant cells perceive stress cues and rapidly produce NO, which in turn directly modulates the activity of specific target proteins via post-translational modifications [8,9]. S-nitrosylation, the covalent modification of specific cysteine residues, constitutes a key mechanism through which cells fine-tune signaling pathways under stress [10]. For example, under high-salinity stress, NO mediates S-nitrosylation of the Cys-374 residue of RGA, a DELLA family protein, thereby inhibiting its degradation and balancing plant growth while enhancing salt tolerance through the antagonism of gibberellin signaling [11]. When shoot apices or leaves perceive stress signals such as high temperature or mechanical wounding, a rapid burst of NO occurs in situ. The NO is then transported over long distances to the roots via the vascular phloem in the form of S-nitrosoglutathione (GSNO) and systemically activates stress-response programs throughout the entire plant, thereby establishing systemic acquired acclimation [12,13,14]. In roots, NO interacts with hydrogen sulfide (H_2_S) to regulate root system architecture through the auxin signaling pathway [15]. Furthermore, NO acts synergistically with hormones such as ethylene (ET) and jasmonic acid (JA) to modulate development in the root apical meristem and elongation zone [16], thereby enhancing plant adaptation to soil salinization. In response to low-temperature stress, NO also functions as a signaling hub. Cold-induced NO accumulation has been shown to positively modulate the inducer of CBF expression (ICE)-C-repeat binding factor (CBF)-cold-regulated (COR) transcriptional cascade, leading to the upregulation of the expression of cold-responsive genes [17]. Studies on the Arabidopsis NO-deficient mutant *nia1nia2noa1-2* confirm that cold-elicited NO accumulation is essential for inducing the expression of both CBF-dependent and CBF-independent cold-responsive genes, such as oxidation-related Zinc Finger 2 (*ZF/OZF2*) [18].

In the context of drought, nitric oxide (NO) also plays crucial roles in plant drought responses [19]. Exogenous NO donors (e.g., sodium nitroprusside, SNP) significantly improve seed germination vigor and promote robust root development, thereby establishing drought resilience [20,21]. NO exerts its regulatory functions through a complex network, activating both cGMP-dependent and cGMP-independent pathways. It interacts with key signaling components, including Ca^2+^, reactive oxygen species (ROS), and mitogen-activated protein kinases (MAPKs), forming an intricate crosstalk network [22,23]. At the post-translational level, NO mediates S-nitrosylation to modulate enzyme activities (e.g., nitrate reductase, NR), creating feedback loops in endogenous NO synthesis and nitrogen metabolism [24,25]. Crucially, NO serves as an important signaling component in ABA-induced stomatal closure. Exogenous NO restores the normal function of ion channels in the *nia1nia2* mutant, promoting stomatal closure and thus reducing leaf water loss under drought stress [26]. Simultaneously, NO synergizes with ABA to regulate stomatal movement, minimizing water loss [27,28].

Stomatal movement, driven by rapid ion fluxes in guard cells, is critical for drought resistance [29]. Ion transport across the plasma membrane alters turgor pressure, directly controlling stomatal aperture [30,31]. The outward-rectifying K^+^ channel GORK mediates K^+^ efflux to reduce turgor and promote closure [32]. ABA activates the BRI1-associated receptor kinase (BAK1), which phosphorylates and stimulates the proton pump PM Arabidopsis H^+^-ATPase isoform 2 (AHA2). Activated AHA2 induces transient H^+^ efflux, causing cytoplasmic alkalinization and subsequent stomatal closure [33]. Additionally, multiple cyclic nucleotide-gated channels (CNGC5/6/9/12) are essential for ABA-specific Ca^2+^ signaling during stomatal regulation [34]. NO critically modulates stomatal movement [35]. Electrophysiological evidence confirms NO influences ion channel activity and membrane potential [36]. However, direct molecular evidence linking NO to specific channels (e.g., KAT1, GORK) and proton pumps (e.g., AHA2) remains scarce. While NO–ROS–ABA crosstalk is established, deeper mechanistic insights into NO’s role in drought resistance are needed [20,37].

At present, the direct downstream targets of NO in regulating drought resistance in cotton remain to be explored. The aim of this study is to identify the direct molecular targets through which NO confers enhanced drought tolerance in cotton, and to dissect how these targets modulate downstream physiological responses to mitigate the adverse effects of drought stress on plant water status and growth, thereby providing excellent targets for crop genetic improvement. Specifically, it demonstrates that exogenous NO treatment significantly enhances drought tolerance in cotton through a synergistic dual-mechanism framework: (i) Antioxidant defense activation occurs, wherein NO upregulates antioxidant enzyme genes (e.g., *SOD*, *CAT*, *POD*) and enhances their activities to scavenge drought-induced ROS, thereby alleviating oxidative damage in leaves; and (ii) Precision stomatal regulation is mediated by site-specific S-nitrosylation of the plasma membrane H^+^-ATPase GhHA2 at Cys329. This post-translational modification stabilizes protein accumulation and facilitates stomatal closure. Consequently, such regulatory events effectively drive stomatal closure and minimize transpirational water loss in plants under drought stress. Collectively, these findings uncover a coordinated, NO-mediated drought resistance mechanism, offering both theoretical foundations and promising practical targets (such as GhHA2-oriented genetic engineering) for genetic improvement of drought-tolerant crops.

## 2. Results

### 2.1. Exogenous NO Enhances Drought Resistance in Cotton

**Figure 1:**
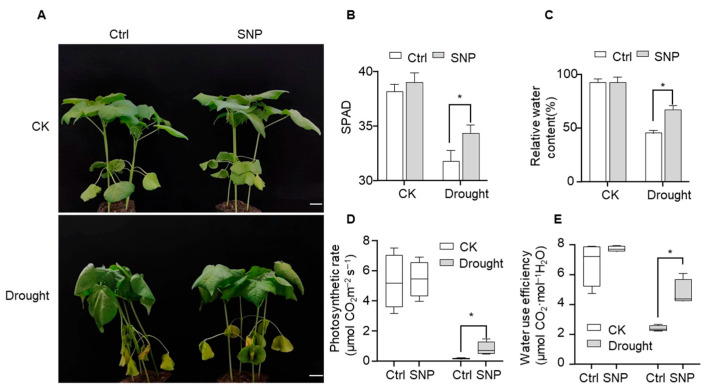
Effects of exogenous SNP application on drought tolerance in cotton seedlings. (**A**) Exogenous application of NO donor SNP to evaluate drought tolerance in cotton seedlings. Two-leaf-stage cotton plants were treated with either H_2_O (Ctrl) or 500 μM SNP. Phenotypes were photographed and recorded after 7 days of drought stress. CK represents well-watered conditions; Drought represents water-deficit conditions. Scale bar = 3 cm. (**B**) SPAD values of cotton leaves under different treatments in Panel (**A**) (* indicates significant difference, *p* < 0.05). The data are shown as means ± SD from four independent repeats (*n* = 4; * indicates significant differences with *p*  <  0.05, Student’s *t*-test). (**C**) Relative water content (RWC) of cotton leaves under different treatments in Panel (**A**). The second true leaf was sampled for RWC measurement upon the appearance of wilting phenotypes (* indicates significant difference, *p* < 0.05). The data are shown as means ± SD from four independent repeats (*n* = 4; * indicates significant differences with *p*  <  0.05, Student’s *t*-test). (**D**) Photosynthetic rate of cotton leaves under different treatments in Panel (**A**). After drought phenotypes emerged, the tested leaf was placed in the leaf chamber of an LI-6400 photosynthesis system, and the photosynthetic rate was recorded upon stabilization. The data are shown as means ± SD from four independent repeats (*n* = 4; * indicates significant differences with *p*  < 0.05, Student’s *t*-test). (**E**) WUE of cotton leaves under different treatments in Panel (**A**). WUE was calculated as follows: WUE = Net photosynthetic rate (Pn)/Transpiration rate (Tr). The data are shown as means ± SD from four independent repeats (*n* = 4; * indicates significant differences with *p*  <  0.05, Student’s *t*-test).

To investigate the effects of NO in the drought tolerance of cotton, we treated cotton plants with exogenous SNP via foliar spraying. Under well-watered conditions (CK), no significant growth differences were observed between the SNP-treated and control plants. However, following drought stress, SNP-treated plants exhibited reduced leaf wilting compared with the control plants (Figure 1A). Consistently, physiological analyses revealed that while chlorophyll content (SPAD values) was comparable between groups under well-watered conditions, the SNP-treated group maintained significantly higher SPAD values under drought stress (Figure 1B). Specifically, the SPAD value of the control group decreased sharply after drought treatment, whereas the SNP-treated group retained a value that was 8% higher than that of the control group This suggests that NO mitigates drought-induced chlorophyll degradation, thereby preserving photosynthetic potential.

Furthermore, drought stress caused a sharp decline in relative leaf water content (RWC) and leaf water potential in control plants. In contrast, SNP-treated plants maintained significantly higher RWC and water potential (Figure 1C and Figure S1). Quantitatively, the SNP-treated group maintained an RWC that was 47% higher and a leaf water potential that was 26% higher than those of the control group. Corroborating this improved water status and chlorophyll retention, photosynthetic rate analysis showed that drought stress nearly abolished photosynthetic capacity in controls, while SNP-treated plants retained a significantly higher rate (Figure 1D). Notably, the photosynthetic rate in the SNP-treated group was 3.5-fold higher than that of the control group.

Additionally, water-use efficiency (WUE) was notably higher in SNP-treated plants under drought conditions (Figure 1E). Specifically, the SNP-treated group maintained a WUE that was twofold higher than that of the control. Collectively, these results demonstrate that exogenous NO significantly alleviates the adverse impacts of drought stress on cotton growth and physiological performance by preserving chlorophyll content, enhancing leaf water retention, maintaining photosynthetic activity, and improving WUE.

### 2.2. NO Enhances ROS Scavenging Capacity and Alleviates Drought-Induced Damage in Cotton

**Figure 2:**
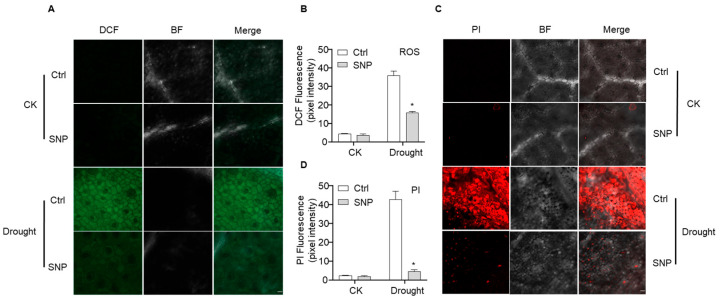
Drought stress-induced ROS accumulation and cell death in cotton leaves. (**A**) The second true leaves of cotton plants under different treatments were selected to detect drought stress-induced ROS accumulation using the ROS probe H_2_DCFDA. Representative images of ROS-specific fluorescence staining in leaf cells were captured using an LSM 900 confocal laser scanning microscope. DCF indicates ROS-specific fluorescence staining, BF represents the bright-field view, and Merge shows the overlay of fluorescence and bright-field images. CK represents well-watered conditions; Drought represents water-deficit conditions; Ctrl represents treatment with H_2_O. Scale bar = 20 μm. (**B**) Quantitative analysis of ROS fluorescence intensity in (**A**). Data were quantified using Image J. * indicates significant differences between groups. The data are shown as means ± SD from three independent repeats (*n* = 3; * indicates significant differences with *p*  <  0.05, Student’s *t*-test). (**C**) The second true leaves of cotton plants under different treatments were stained with propidium iodide (PI) to detect drought stress-induced cell death. Representative images of PI-stained leaf cells were captured using an LSM 900 confocal laser scanning microscope. PI indicates cell death-specific staining, BF represents the bright-field view, and Merge shows the overlay of fluorescence and bright-field images. Scale bar = 20 μm. (**D**) Quantitative analysis of PI fluorescence intensity in (**C**). Data were quantified using Image J. The data are shown as means ± SD from three independent repeats (*n* = 3; * indicates significant differences with *p*  <  0.05, Student’s *t*-test).

Drought stress triggers excessive accumulation of ROS and causes cellular damage in plants. To explore the regulatory effects of exogenous NO on ROS scavenging and cell integrity in cotton under drought, this study performed in vivo tissue staining combined with confocal microscopy. Under well-watered conditions, no obvious ROS accumulation was observed in either the control or SNP-treated groups (Figure 2A,B), and propidium iodide (PI) staining for cell viability showed no significant difference (Figure 2C,D). Under drought stress, however, the control exhibited a substantial ROS burst and severe cell death, characterized by sharply increased ROS and PI fluorescence intensities. In contrast, DCF and PI fluorescence were significantly reduced by 55.8% and 89.6%, respectively, in the SNP-treated group compared with the drought-stressed control (Figure 2), indicating that exogenous NO effectively scavenges drought-induced excessive ROS, mitigates oxidative stress and cellular damage, and preserves cell structural stability.

### 2.3. Exogenous NO Enhances ROS Scavenging Capacity by Upregulating the Expression Levels of Endogenous Antioxidant Enzyme Genes in Cotton

**Figure 3:**
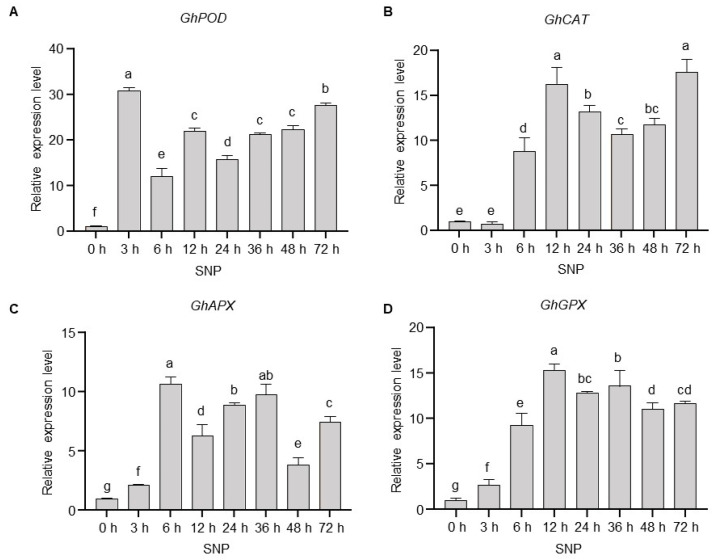
Time-course expression profiles of antioxidant enzyme genes (*GhPOD*, *GhCAT*, *GhAPX*, *GhGPX*) in response to SNP treatment. (**A**–**D**) Relative expression levels of *GhPOD* (**A**), *GhCAT* (**B**), *GhAPX* (**C**), and *GhGPX* (**D**) genes at different time points (0 h, 3 h, 6 h, 12 h, 24 h, 36 h, 48 h, 72 h) after SNP treatment, as determined by quantitative real-time PCR (qPCR). Data are presented as relative expression values and are shown with the mean ± SD from three independent repeats (*n* = 3; different letters indicate significant differences with *p*  <  0.05, based on one-way ANOVA and Duncan/LSD multiple comparisons).

S-nitrosoproteomic analysis revealed that four key antioxidant enzymes in *Gossypium hirsutum*, namely, glutathione peroxidase, catalase, peroxidase, and L-ascorbate peroxidase, undergo S-nitrosylation upon SNP treatment (Table S1). All modified residues were identified as cysteines. Notably, both catalase and peroxidase exhibited multi-site S-nitrosylation, suggesting that nitric oxide can directly target and regulate core antioxidant enzymes via this post-translational modification, thereby modulating the ROS-scavenging machinery in cotton. To investigate the regulatory effect of nitric oxide on these enzymes, we first examined the temporal expression profiles of *GhCAT*, *GhAPX, GhPOD*, and *GhGPX* in cotton seedlings treated with the NO donor SNP. Our findings revealed that all four genes underwent significant time-dependent upregulation, albeit with distinct response kinetics. Notably, *GhPOD* exhibited the most rapid response to NO signaling, showing marked upregulation within 3 h post treatment, which was 30.8-fold higher than that at 0 h (Figure 3A), which suggests its pivotal role in activating early-phase antioxidant defense. In contrast, *GhCAT* displayed a biphasic expression pattern characterized by two prominent peaks at 12 h and 72 h, reaching 16.2-fold and 17.5-fold relative to the 0 h level, respectively. This pattern, defined by an initial upregulation, followed by a transient decline, and a subsequent late-phase re-enhancement (Figure 3B), underscores the functional importance of GhCAT in sustaining long-term antioxidant capacity. In addition, the expression of *GhAPX* and *GhGPX* peaked at 6 h and 12 h after SNP treatment, with the expression levels being 10.7-fold and 15.1-fold of the 0 h group, respectively (Figure 3C,D).

The temporally coordinated responses of these genes demonstrate that exogenous NO induces antioxidant gene expression through a phased regulatory mechanism: (a) a rapid initial response dominated by *GhPOD* (Figure 3A); (b) an intermediate maintenance phase coordinated by *GhAPX* and *GhGPX* (Figure 3C,D); and (c) a late-phase reinforcement driven by *GhCAT* upregulation (Figure 3B). This sequential, multigene synergy systematically enhances the ROS scavenging capacity of cotton at the transcriptional level. Collectively, our results indicate that NO establishes a robust oxidative defense system by sustaining high expression levels of endogenous antioxidant enzymes, thereby mitigating drought-induced oxidative damage. This strategy of temporally partitioned gene activation suggests an evolutionarily optimized defense cascade, wherein rapid-response and long-term maintenance mechanisms are molecularly segregated to maximize stress protection.

### 2.4. Exogenous NO Enhances ROS Scavenging Capacity by Affecting Antioxidant Enzyme Activities in Cotton

**Figure 4:**
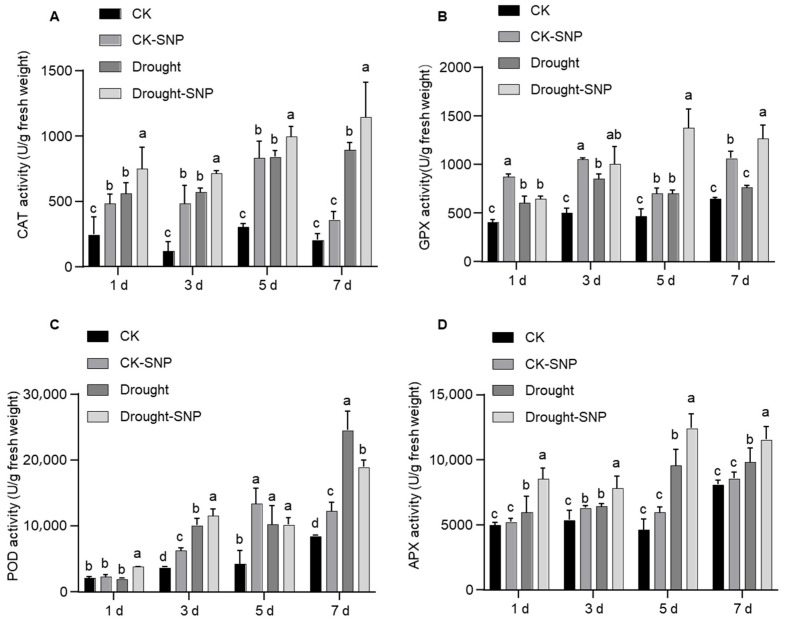
Effects of exogenous SNP on antioxidant enzyme activities (CAT, GPX, POD, APX) under drought stress and well-watered conditions. (**A**–**D**) Enzyme activities of CAT (**A**), GPX (**B**), POD (**C**), and APX (**D**) measured at 1 d, 3 d, 5 d, and 7 d under four treatment groups: Well-watered + water spray (CK); Well-watered + SNP spray (CK-SNP); Drought stress + water spray (Drought); Drought stress + SNP spray (Drought-SNP). Enzyme activities are expressed in units per gram fresh weight (U/g FW). Data are shown with the mean ± SD from three independent repeats (*n* = 3; different letters indicate significant differences with *p*  <  0.05, based on one-way ANOVA and Duncan/LSD multiple comparisons).

To further verify the physiological basis of NO-mediated ROS scavenging, we measured dynamic changes in the activities of four core antioxidant enzymes under different treatments. They are catalase (CAT), glutathione peroxidase (GPX), peroxidase (POD), and ascorbate peroxidase (APX). Under well-watered conditions, especially at 3 days after treatment, SNP significantly increased the activities of all four enzymes (Figure 4A–D). Drought stress markedly induced the activities of all four enzymes. From 1 d to 7 d of stress, CAT, GPX, POD, and APX activities in drought-treated control plants were significantly higher than under well-watered conditions (Figure 4A–D), reflecting the intrinsic antioxidative defense response of cotton to drought. Exogenous SNP further amplified this response under drought: CAT activity was 18–28% higher in the SNP group than in the Drought group at 5 d and 7 d, and 3.3- to 4.5-fold higher than in the CK group (Figure 4A). GPX activity was 57–70% higher than in the Drought group and 1.5- to 2.7-fold higher than in the CK group (Figure 4B). APX activity was 30% higher than in the Drought group at 5 d and 2.7-fold higher than in CK; it decreased slightly at 7 d but remained 18% higher than in the Drought group and 1.4-fold higher than in CK (Figure 4D). POD activity in SNP-treated plants was significantly higher than in the Drought group during early drought (1 d and 3 d), reaching a 92% increase (nearly 2-fold) at 1 d (Figure 4C). Although slightly lower than in the Drought group at 7 d, POD activity was still 2.2-fold higher than in CK and remained elevated overall.

In summary, exogenous NO further elevates antioxidant enzyme activities under drought, establishing a highly efficient ROS-scavenging system. These results confirm at the enzymatic level that enhanced NO-mediated antioxidative defense represents a core physiological mechanism by which NO mitigates drought-induced oxidative damage and improves drought tolerance in cotton.

### 2.5. Exogenous NO Enhances Drought Tolerance in Cotton by Promoting Stomatal Closure

**Figure 5:**
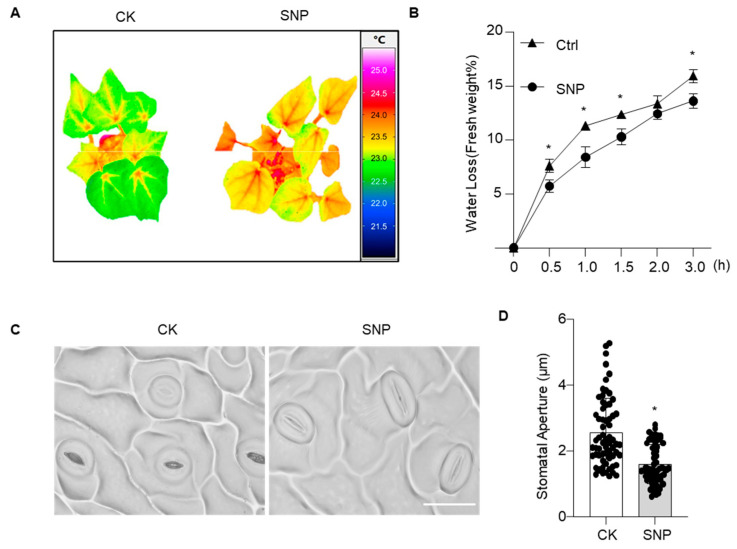
Water loss and stomatal responses of cotton plants to exogenous SNP treatment. (**A**) Infrared thermal imaging analysis of control (CK, water spray) and SNP-treated plants under well-watered conditions. Cotton plants were photographed using an infrared camera, and Image processing was performed using infrared imaging software (IRBIS 3) (**B**) Water loss rate measurements for different treatments shown in panel (**A**). The aerial parts of cotton plants were subjected to dehydration treatment. Fresh weight loss percentage was calculated after 0, 0.5, 1.0, 1.5, 2.0, and 3.0 h of treatment. The data are shown as means ± SD from three independent repeats (*n* = 3; * indicates significant differences with *p*  <  0.05, Student’s *t*-test). (**C**) Stomatal morphology observations under different treatments from panel (**A**). Stomata on the abaxial leaf epidermis were visualized using nail polish imprints. Scale bar = 50 μm. (**D**) Statistical analysis of stomatal aperture corresponding to panel (**C**). Box plots show the distribution of stomatal aperture in CK and SNP-treated groups. Data are shown with the mean ± SD from three independent repeats (*n* = 3), with at least 60 stomata quantified (* indicates significant differences with *p*  <  0.05, Student’s *t*-test).

To reveal the water regulatory mechanism underlying NO-enhanced drought tolerance, infrared thermal imaging was first performed. Under well-watered conditions, leaf temperature was significantly higher in SNP-treated cotton plants than in controls, with colors shifted toward the high-temperature range (Figure 5A), indicating reduced transpiration water loss. Further measurement of the excised leaf water loss rate showed that, over 0–3.0 h, the water loss rate was consistently significantly higher in control leaves than in SNP-treated leaves at 1.0 h after excision, the water loss rate of control leaves was 25.7% higher than that of SNP-treated leaves (Figure 5B), confirming that NO effectively reduces water loss in cotton leaves.

As stomata are the primary gateways for transpiration and critical determinants of drought resistance, we observed stomatal phenotypes and measured aperture under SNP treatment (Figure 5C,D). SNP treatment significantly reduced stomatal aperture in cotton leaves, demonstrating that exogenous NO reduces transpiration by promoting stomatal closure. Taken together, these findings indicate that NO induces rapid stomatal closure, decreases transpiration rate, reduces water loss, and thereby maintains water homeostasis in cotton plants.

### 2.6. GhHA2 Is Regulated by NO-Mediated S-Nitrosylation Modification

Drought-induced stomatal closure is a key mechanism in plant drought stress responses [38]. However, the precise mechanism by which NO regulates stomatal movement through molecular targets remains incompletely understood. Studies have shown that NO regulates the function of multiple guard cell signaling proteins via S-nitrosylation, thereby modulating stomatal closure [22]. The activity of plasma membrane cation channels in guard cells also plays an essential role during stomatal closure [39]. In Arabidopsis, the plasma membrane H^+^-ATPase isoform AHA2 is known to regulate stomatal closure. Notably, S-nitrosylation of H^+^-ATPase2 in tomato roots affects plant salt tolerance [40]. However, whether H^+^-ATPase acts as a downstream target of NO to regulate stomatal movement under drought in cotton remains unclear.

**Figure 6:**
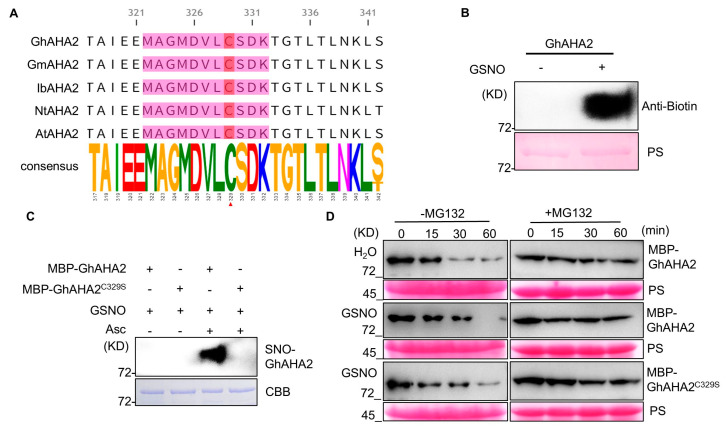
Conservation, S-nitrosylation, and protein stability of Cys329 in GhHA2, a key regulatory site in cotton. (**A**) The Cys329 residue in GhHA2 is evolutionarily conserved. Multiple-sequence alignment of AHA2 proteins from cotton (GhHA2), soybean (GmHA2), sweet potato (IbHA2), tobacco (NtHA2), and Arabidopsis (AHA2) reveals conserved Cys329 (red arrow, corresponding to GhHA2^Cys329^), a putative S-nitrosylation site. Different colors indicate amino acid properties, while letter height reflects sequence conservation. (**B**) In vitro S-nitrosylation assay of GhHA2. Biotin-switch assay detects S-nitrosylated GhHA2 after GSNO treatment, with anti-biotin immunoblot showing S-nitrosylation signals. Ponceau S (PS) staining verifies equal loading. (**C**) Cys329 is essential for GhHA2 S-nitrosylation. Comparative analysis of S-nitrosylation levels between wild-type MBP-GhHA2 and the MBP-GhHA2^C329S^ mutant. Coomassie Brilliant Blue (CBB) staining confirms equal protein loading. (**D**) S-nitrosylation regulates GhHA2 protein stability in cell-free degradation assays. Time-course (0–60 min) analysis of MBP-GhHA2^WT^ and MBP-GhHA2^C329S^ stability under H_2_O or GSNO treatment, with MG132 (proteasome inhibitor) as control. Protein levels were detected by anti-MBP immunoblot, with PS staining showing loading controls.

Accordingly, we identified a cotton homolog of plasma membrane H^+^-ATPase2 via BLAST (SequenceServer 2.0.0) analysis and named it *Gossypium hirsutum* PM H^+^-ATPase isoform 2 (GhHA2). GPS-SNO 1.0 software predicted Cys329 as a putative S-nitrosylation site. Multi-species sequence alignment revealed that GhHA2 is highly homologous to HA2 from soybean, sweet potato, tobacco, and Arabidopsis, with a highly conserved motif at the Cys329 residue (Figure 6A). Due to difficulties in full-length prokaryotic expression of transmembrane proteins, we purified a partial GhHA2 fusion protein for in vitro S-nitrosylation assays. After treatment with the NO donor GSNO, the GhHA2 protein was S-nitrosylated (Figure 6B), verifying that GhHA2 is a direct target of NO-mediated S-nitrosylation.

To determine whether Cys329 is essential for NO-induced S-nitrosylation, site-directed mutagenesis was performed. When Cys329 was mutated to serine (C329S), GSNO-induced S-nitrosylation was nearly abolished (Figure 6C), confirming Cys329 as the key site for NO-mediated S-nitrosylation of GhHA2. To explore the effect of S-nitrosylation on the GhHA2 protein, we analyzed protein stability using a cell-free system (Figure 6D). Under control (H_2_O) treatment, wild-type MBP-GhHA2 gradually degraded over time. GSNO treatment significantly delayed degradation of wild-type MBP-GhHA2, whereas the C329S mutant was unaffected. Moreover, the proteasome inhibitor MG132 stabilized protein abundance, indicating that NO-mediated S-nitrosylation at Cys329 enhances GhHA2 stability by inhibiting proteasomal degradation. In conclusion, GhHA2 is a direct target of NO. NO stabilizes the GhHA2 protein via S-nitrosylation at Cys329, thereby participating in stomatal movement and water homeostasis, providing a pivotal molecular mechanism for NO-enhanced drought tolerance in cotton.

### 2.7. GhHA2 Positively Regulates Drought Tolerance in Cotton

**Figure 7:**
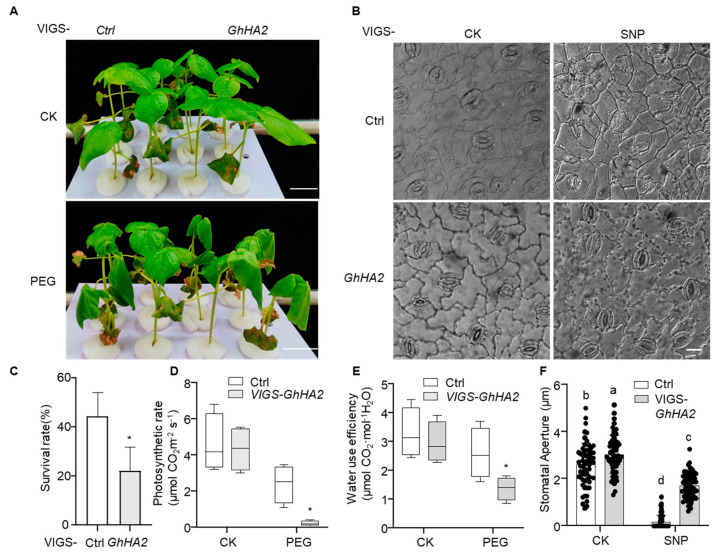
VIGS-*GhHA2* cotton seedlings response to PEG-induced drought stress or SNP treatment. (**A**) VIGS-*GhHA2* cotton exhibits heightened sensitivity to PEG-mediated drought stress. Hydroponically grown VIGS-*GhHA2* seedlings (12 plants per culture plate) were subjected to PEG treatment at the two-true-leaf stage. Phenotypes were documented post treatment. CK represents normal hydroponic conditions without PEG treatment, while PEG represents drought stress simulated by PEG6000 treatment. VIGS-*Ctrl* represents VIGS-*GFP*. Scale bar = 3 cm. (**B**) Stomatal morphology on abaxial leaf surfaces of VIGS-*GhHA2* cotton under different treatments. Excised leaves were exposed to either water or SNP for 3 h before imaging. Scale bar = 20 μm. (**C**) Survival rates of VIGS-*GhHA2* cotton from panel (**A**). * denotes statistically significant differences between groups (*p* < 0.05). Data are shown with the mean ± SD from three independent repeats (*n* = 3; * indicates significant differences with *p*  <  0.05, Student’s *t*-test). (**D**,**E**) Photosynthetic rate and water-use efficiency (WUE) measurements of VIGS-*GhHA2* cotton under control and PEG treatments from panel (**A**). Net photosynthetic rates were determined using an LI-6400 photosynthesis system, with WUE calculated accordingly. * indicates significant intergroup differences (*p* < 0.05). Data are shown with the mean ± SD from four independent repeats (*n* = 4; * indicates significant differences with *p*  <  0.05, Student’s *t*-test). (**F**) Quantification of stomatal aperture in control (VIGS-*Ctrl*) and VIGS-*GhHA2* plants from panel (**B**). Apertures were measured using Image J. Data are shown with the mean ± SD from three independent repeats (*n* = 3), with at least 60 stomata quantified. Different letters indicate significant differences with *p*  <  0.05, based on one-way ANOVA and Duncan/LSD multiple comparisons.

To clarify the function of GhHA2 in cotton drought tolerance and its regulatory role in NO signaling, we first examined the effect of exogenous SNP on hydroponic cotton seedlings under PEG-simulated drought. NO treatment enhanced drought resistance and reduced the leaf wilting rate (Supplementary Figure S3A,B). We then silenced *GhHA2* using virus-induced gene silencing (VIGS) and verified silencing efficiency by qRT-PCR (Figure S2). Phenotypic and physiological responses to PEG-simulated drought were then evaluated. Under normal conditions, the VIGS-*Ctrl* and VIGS-*GhHA2* groups showed no significant growth differences. After drought treatment, VIGS-*GhHA2* plants exhibited more severe wilting (Figure 7A) and approximately a 50% reduction in survival rate compared with controls (Figure 7C).

Physiological analysis further showed that under drought, both the photosynthetic rate and WUE were significantly lower in VIGS-*GhHA2* plants than in controls (Figure 7D,E). Under CK conditions, photosynthetic rates were comparable between VIGS-*Ctrl* and VIGS-*GhHA2* plants. After PEG treatment, the photosynthetic rate of VIGS-*Ctrl* plants remained relatively stable, whereas that of VIGS-*GhHA2* plants decreased sharply, dropping to only 8% of the control level. Similarly, while WUE was comparable between genotypes under CK conditions, PEG treatment significantly reduced the WUE of VIGS-*GhHA2* plants to approximately 54% of the control. These results indicate that GhHA2 positively regulates drought tolerance and that its silencing impairs photosynthetic carbon assimilation and WUE. To test stomatal responsiveness to NO, we examined excised leaves microscopically (Figure 7B,F). Under normal conditions, VIGS-*GhHA2* plants displayed larger stomatal apertures. Following SNP treatment, stomatal aperture decreased markedly in control plants, but only partially in VIGS-*GhHA2* plants, which showed impaired responsiveness to NO. These results demonstrate that GhHA2 is a key downstream component required for NO-induced stomatal closure, and its silencing impairs NO-mediated stomatal closure, exacerbating water loss under drought.

## 3. Discussion

### 3.1. Dual-Pathway Coordinated Regulatory Mechanism of NO-Mediated Drought Response in Cotton

**Figure 8:**
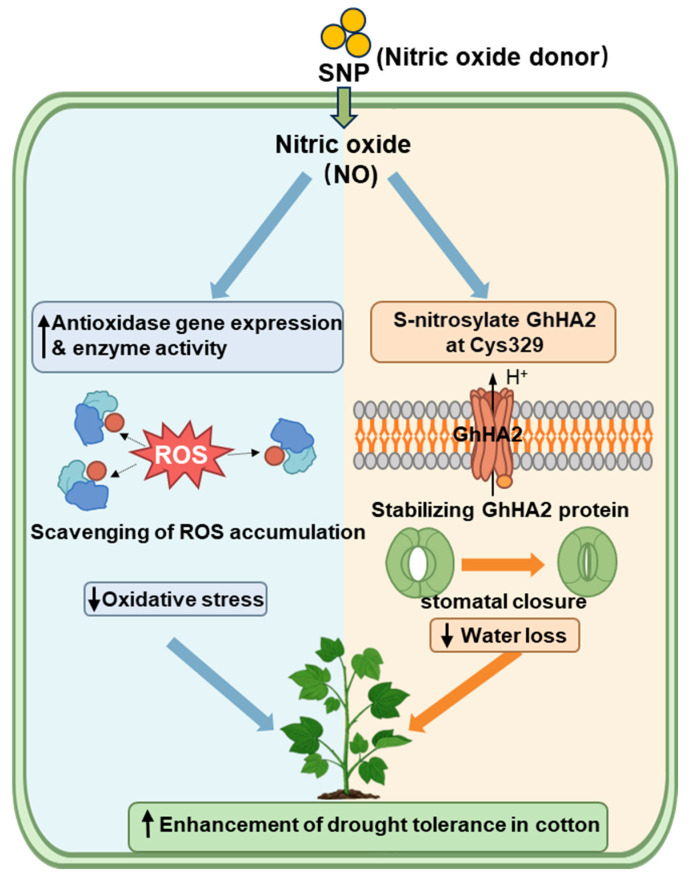
Molecular mechanism of NO-mediated drought resistance in cotton.

This study systematically elucidates the molecular regulatory network by which the exogenous NO donor, SNP, enhances drought resistance in cotton through two synergistic pathways. First, NO upregulates the expression and activity of antioxidant enzymes to directly scavenge drought-induced ROS, thereby suppressing oxidative stress and mitigating cellular damage and membrane disruption. Concurrently, via S-nitrosylation at the Cys329 residue of the plasma membrane H^+^-ATPase GhHA2, NO stabilizes the protein, enhancing its capacity to regulate stomatal movement. Consequently, this dual mechanism promotes stomatal closure to minimize water loss while maintaining cellular homeostasis. Ultimately, the coordinated action of these complementary pathways—encompassing both ROS scavenging and hydraulic regulation—collectively fortifies cotton’s drought tolerance (Figure 8).

NO enhances cotton drought resistance through two synergistic pathways: Antioxidant Pathway and Water Homeostasis Regulation Pathway. On the one hand, NO upregulates the expression of antioxidant enzyme genes and enhances their activity, effectively scavenging drought-induced ROS accumulation. This alleviates oxidative stress and mitigates cellular damage. On the other hand, NO mediates S-nitrosylation at Cys329 of GhHA2, which stabilizes the GhHA2 protein. This post-translational modification promotes stomatal closure, reduces water loss under drought stress, and maintains cellular water homeostasis. Through the coordinated action of these two pathways, NO ultimately enhances cotton’s drought resistance capacity.

### 3.2. NO-Mediated Antioxidant Defense in Cotton Involves Transcriptional and Enzymatic Coordination

Drought stress triggers NO accumulation, and NO acts as a pivotal dual-signaling messenger in plant drought response [41]. Plants possess a sophisticated antioxidant system to detoxify ROS, in which antioxidant enzymes such as POD and CAT play essential roles [42]. Our results demonstrated that exogenous NO treatment enhanced antioxidant enzyme activities, eliminating excessive ROS (Figure 2A,B) and thereby alleviating drought-induced cell death and plasma membrane damage in cotton (Figure 2C,D). Previous studies in soybean have shown that NO activates antioxidant systems, as evidenced by significant increases in antioxidant enzyme activities; however, the temporal dynamics of these enzyme activities were not analyzed [43]. Notably, our findings revealed that NO upregulates the expression of core antioxidant enzyme genes (*GhCAT*, *GhAPX*, *GhPOD*, and *GhGPX*) in cotton with distinct temporal expression patterns (Figure 3). Transcriptional profiling indicated that *GhPOD* is the earliest NO-responsive gene and showed distinct temporal regulatory dynamics. Although transcript analysis confirmed that *GhPOD* is the primary early NO-responsive gene, POD enzymatic activity decreased at the late stage of drought stress. Submergence stress studies in wheat have shown that the strong induction of peroxidase genes serves as an early biomarker for waterlogging tolerance in specific cultivars [44]. Given that NO functions as a conserved early signaling molecule in response to multiple abiotic stresses including drought and salinity [45], we hypothesize that analogous signaling logic is conserved in cotton drought responses. Specifically, the rapid induction of *GhPOD* under drought stress is closely associated with NO-mediated early signaling events. It is well established that stress signals rapidly induce a NO burst, which further modulates secondary messengers such as cGMP-dependent Ca^2+^ influx, thereby activating Ca^2+^/CaM-regulated transcriptional modules, such as calmodulin-binding transcription activators (CAMTAs), and initiating the expression of downstream stress-responsive genes [46,47]. Meanwhile, NO directly modulates key transcription factors including myeloblastosis 2 (MYB2) and ABA insensitive 5 (ABI5) via S-nitrosylation to synergistically regulate downstream stress-related genes [10]. These parallel and synergistic early signaling pathways provide a robust mechanistic framework for explaining the rapid transcriptional activation of *GhPOD* under drought conditions.

In contrast, *GhCAT* exhibited a marked biphasic induction pattern (Figure 3), accompanied by a significant time-dependent increase in CAT activity (Figure 4), which efficiently mitigated drought-triggered oxidative damage via ROS scavenging. The biphasic activation of *GhCAT* suggests the existence of a feedforward amplification loop. Its initial activation may be triggered by the aforementioned NO-derived early signals, while the subsequent activation is potentially maintained by ROS-triggered mitogen-activated protein kinase (MAPK) cascades, which rely on continuous stress perception and signal transduction [48]. Consistent with the above results, *GhPOD* was identified as the earliest NO-responsive gene at the transcriptional level, whereas POD enzymatic activity decreased in the late drought stage. Importantly, our S-nitrosoproteomic analysis independently identified four antioxidant enzymes (*GhCAT*, *GhAPX*, *GhPOD*, and *GhGPX*) as potential substrates for NO-mediated S-nitrosylation (Table S1). Of particular interest, two critical S-nitrosylation sites were identified in the protein encoded by *GhPOD*. Accumulating evidence has shown that NO-mediated post-translational modifications exert dual regulatory effects (activation or inhibition) on enzyme activities [49]. Accordingly, we propose that the dynamic changes in POD activity at different drought stages are mediated by distinct S-nitrosylation sites, and the late-stage reduction in POD activity is largely attributable to enzyme inactivation caused by NO-induced S-nitrosylation.

Taken together, under drought stress, NO may exert reversible and dynamic regulation on multiple antioxidant enzymes through post-translational modifications such as S-nitrosylation, thereby achieving fine control of intracellular ROS scavenging. This regulation can rapidly activate the antioxidant defense system when oxidative stress intensifies, while preventing excessive and sustained activation of antioxidant enzymes after stress is alleviated, thus maintaining an effective balance of cellular redox homeostasis. A recent multi-species meta-analysis has confirmed that the enhancing effect of NO on antioxidant defense systems shows cross-species universality [50]. This strongly supports our finding that NO-mediated dynamic regulation of antioxidant enzymes in cotton represents an important and conserved adaptive mechanism for crops to cope with drought stress.

### 3.3. S-Nitrosylation of GhHA2 Mediates Stomatal Closure to Enhance Drought Tolerance in Cotton

Stomata, serving as the primary gateways for plant transpiration, play a pivotal role in drought resistance [51]. Research has established NO as a crucial signaling molecule regulating stomatal closure [52]. Given that NO inhibits ABA-mediated K^+^ influx but exerts no significant effect on ABA-induced K^+^ efflux [36], we investigated plasma membrane ion channel proteins directly involved in stomatal movement. Protein S-nitrosylation, a key post-translational modification, serves as the primary signaling mechanism for NO, critically regulating plant hormone signaling, development, and stress responses [24,53]. Since NO had a negligible effect on K^+^ efflux, we first excluded the outward-rectifying K^+^ channel GORK. Due to functional redundancy among CNGC family members, we subsequently performed in silico predictions of S-nitrosylation sites using GPS-SNO software for both the inward-rectifying K^+^ channel GhKAT1 and the plasma membrane H^+^-ATPase GhHA2, selecting the highest-scoring sites for in vitro validation. Our results demonstrate that Cys329 of GhHA2 serves as a direct target for NO-mediated S-nitrosylation (Figure 6), while GhKAT1 unexpectedly showed no responsiveness to this modification (Figure S4). Silencing GhHA2 resulted in partial insensitivity to NO-induced stomatal closure (Figure 7B,F), positioning GhHA2 downstream of NO signaling. In *Arabidopsis*, the *aha2* mutant exhibits increased sensitivity to drought stress [33]. At the molecular level, protein stability directly determines the intracellular abundance and activity of H^+^-ATPase [54]. Previous studies have demonstrated that NO can protect substrate proteins from recognition and degradation by the 26S proteasome through S-nitrosylation, thereby enhancing their stability and ensuring efficient accumulation of target proteins in cells [55]. Intriguingly, our study revealed that NO-mediated S-nitrosylation of GhHA2 enhances its protein stability (Figure 6D). Based on these findings, we hypothesize that in cotton guard cells, NO-triggered S-nitrosylation inhibits the ubiquitination of GhHA2, thereby reducing its proteasomal degradation. This ultimately sustains H^+^-ATPase protein levels in guard cells and ensures drought stress-induced stomatal closure.

Analysis of the relative expression levels of *GhHA2* and *GhKAT1* under SNP treatment revealed distinct temporal dynamics. The expression of *GhHA2* was rapidly and transiently upregulated, peaking at 12 h post treatment (5.0-fold relative to the 0 h) and then declining significantly (Figure S5A). In contrast, *GhKAT1* showed delayed but sustained upregulation, reaching a maximum at 24 h (6.5-fold relative to 0 h), with relatively high transcript levels still persisting at 48 h (4.4-fold relative to 0 h) (Figure S5B). These differential transcriptional patterns indicate that *GhHA2* acts as an early response gene in the NO signaling pathway. Numerous studies have demonstrated that NO functions as a pivotal second messenger in the ABA signaling cascade. As a plasma membrane H^+^-ATPase, AHA2 plays a critical positive regulatory role in early ABA signal transduction [33]. Accordingly, the rapid transcriptional induction of *GhHA2* by NO may quickly elevate its mRNA abundance, providing a pool of transcripts for the subsequent synthesis and accumulation of AHA2 protein and thereby ensuring sufficient H^+^-ATPase abundance in guard cells. Our analysis of NO-mediated post-translational modification of GhHA2 further indicates that NO dually targets and regulates this key proton pump in cotton guard cells through transcriptional modulation and S-nitrosylation. This dual regulation coordinately maintains the protein abundance and functional stability of GhHA2, thereby mediating stomatal closure and drought tolerance under drought stress.

KAT1 is the predominant voltage-gated inwardly rectifying K^+^ channel responsible for K^+^ influx in guard cells, and KAT1-mediated K^+^ influx constitutes a core driving force for stomatal opening [31,56]. Under drought stress, ABA signaling represses KAT1 function through multiple parallel negative regulatory mechanisms, including the promotion of selective endocytosis and recycling of KAT1 protein, which dynamically modulates the abundance of functional channels at the plasma membrane [57]. The delayed and sustained NO-induced transcriptional upregulation of *GhKAT1* observed in this study suggests that GhKAT1 is primarily involved in regulating late stress responses or post-stress recovery processes, thus laying a molecular foundation for stomatal reopening. Notably, the dynamic transcriptional regulation of *KAT1* remains poorly understood [56,58]. The NO-dependent differential transcriptional regulation uncovered in this study offers a novel perspective for understanding the multi-level synergistic regulation of ion transporters in guard cells. Further investigations are required to elucidate the specific signaling pathways underlying these regulatory processes.

Wei et al. [40] recently reported that under saline–alkali stress, S-nitrosylation of the Cys206 residue in tomato root H^+^-ATPase 2 (HA2) negatively regulates its enzymatic activity, whereas melatonin alleviates nitrosative damage and maintains HA2 function by reducing this modification. In stark contrast, our study reveals that under drought stress, S-nitrosylation of the Cys329 residue in cotton GhHA2 positively regulates protein stability, thereby promoting stomatal closure. This discrepancy in regulatory modes may be attributed to two main factors. First, the physiological role of NO is highly context-dependent: excessive NO accumulation under saline–alkali stress leads to nitrosative damage, whereas under drought stress, NO functions primarily as a protective signaling molecule. Second, there is likely a tissue-specific functional requirement for H^+^-ATPase; roots require sustained pump activity for ion homeostasis, while guard cells may prioritize mechanisms that facilitate stomatal closure. Furthermore, S-nitrosylation at Cys206 and Cys329 likely mediates distinct functional outcomes.

Collectively, direct molecular evidence for how NO regulates stomatal movement via post-translational modification of target proteins has long been lacking. In this study, we identified GhHA2 as a key direct target of NO in stomatal closure and, for the first time in cotton, verified that the plasma membrane H^+^-ATPase GhHA2 functions as a core downstream target of NO signaling in controlling stomatal dynamics. Our work elucidates a novel mechanism whereby S-nitrosylation stabilizes the H^+^-ATPase protein by inhibiting proteasomal degradation, subsequently driving stomatal closure. These finding significantly expand the current understanding of the post-translational regulatory network of H^+^-ATPases and provide novel and valuable target genes for the genetic improvement of drought-resistant cotton. This study raises the intriguing possibility that NO may finely regulate the expression of channel proteins through dynamic transcriptional regulatory mechanisms, providing a new direction for future research.

### 3.4. Limitations and Future Perspectives

Our study systematically elucidates how exogenous NO enhances cotton drought resistance through a coordinated regulatory network involving antioxidant defense, water homeostasis maintenance, and post-translational modifications of key functional proteins. However, several limitations warrant further investigation: First, although we adhered to established methods by employing the exogenous NO donor SNP to induce NO accumulation [59,60], our study is constrained by a lack of genetic resources to resolve the spatiotemporal dynamics of endogenous NO production during drought stress. To address this limitation and validate our observations, future investigations should leverage genetic tools to precisely manipulate endogenous NO levels. Second, although our in vitro S-nitrosylation assays identified GhHA2 as a NO target, the functional consequences of this modification require further characterization. The current absence of transgenic cotton lines (for overexpression or knockout) prevents in vivo validation of GhHA2 S-nitrosylation. Generating stable transgenic lines will be essential for comprehensively assessing GhHA2’s role in drought resistance.

Notably, while field trials by Akin et al. [61] demonstrated that combined NO and Asn treatment enhances cotton yield, the underlying molecular mechanisms remain unexplored. Our findings establish a foundation for future research on improving drought resistance in cotton and other crops. Subsequent field studies should evaluate the ultimate impact on cotton yield. The identification of GhHA2 as a key NO signaling target provides a promising candidate for genetic engineering, potentially enabling development of cotton varieties with superior stomatal regulation and drought resistance. Furthermore, our discovery of temporal regulation in antioxidant enzymes suggests that optimizing the timing of NO application could maximize its protective effects.

## 4. Materials and Methods

### 4.1. Plant Materials and Growth Conditions

In this study, the conventional upland cotton variety Xinshi 17 (*Gossypium hirsutum* L.) was used. For the soil culture experiment, nutrient soil was employed for seedling cultivation. Cotton seeds were disinfected with 3% H_2_O_2_ for 20 min and then rinsed three times with sterile water. The seeds were soaked in the dark at 28 °C overnight, and seeds with consistent germination were selected and sown in nutrient pots (8.5 × 8.5 × 9.5 cm) containing 140 g of nutrient soil, with four seeds per pot. Only uniformly growing cotton seedlings were retained. For the hydroponic experiment, fine sand was used for seedling cultivation, and cotton seeds with consistent germination were sown in the sand. After the seed coats were shed and the cotyledons were fully expanded, uniformly growing seedlings were selected and transferred to Hoagland nutrient solution until the development of two true leaves. All materials were cultivated in a plant growth chamber with a light intensity of 250 ± 50 μmol m^−2^·s^−1^, an average humidity of 40–60%, and a photoperiod of 14 h of light/10 h of darkness to ensure normal growth of the cotton seedlings.

### 4.2. Antioxidant Enzyme Activity Assays

Fresh leaf tissue (0.1 g) was weighed and homogenized in 1 mL of the corresponding extraction buffer on ice. The homogenate was centrifuged at 4 °C for 10 min, and the supernatant was collected and kept on ice for subsequent assays. Catalase (CAT) activity was measured using a CAT activity assay kit (Solarbio, Beijing, China). Ascorbate peroxidase (APX) activity was determined using a CheKine™ APX activity assay kit (Abbkine, Wuhan, China). Peroxidase (POD) activity was measured using a POD activity assay kit (Solarbio), and glutathione peroxidase (GSH-Px/GPX) activity was assessed using a GPX activity assay kit (Solarbio). The assay methods followed the instructions provided in the respective kit manuals. All experiments were performed with three independent biological replicates (*n* = 3). The data are shown as means ± SD.

### 4.3. Prokaryotic Expression and Purification of MBP-GhHA2 and His-KAT1 Proteins

The recombinant plasmids pMAL-*GhHA2*, pMAL-*GhHA2^C329S^* and pET28a-*GhKAT1* were separately transformed into *Escherichia coli* BL21 competent cells. After overnight incubation at 37 °C, single positive colonies were picked and inoculated into LB liquid medium containing the appropriate antibiotic. The cultures were grown at 37 °C with shaking at 200 rpm until the OD_600_ reached 1.0. The culture was then transferred to a shaker pre-cooled to 16 °C and allowed to equilibrate. Protein expression was induced by adding isopropyl β-D-1-thiogalactopyranoside (IPTG) to a final concentration of 0.2 mM, followed by incubation overnight at 16 °C with shaking at 120 rpm. The bacterial cells were harvested by centrifugation at 4 °C and 4000 rpm for 15 min. The cell pellet was resuspended in 10 mL of protein lysis buffer, and lysozyme was added to a final concentration of 100 µg/mL. After thorough mixing, the suspension was incubated on ice for 15 min. The tube containing the resuspended cells was placed in an ice-water bath, and the cells were disrupted by sonication using an ultrasonic cell disruptor (Ningbo Scientz Biotechnology Co., Ltd., Ningbo, China). The lysate was then centrifuged at 4 °C and 12,000 rpm for 20 min. The clarified supernatant was transferred to a sterile 15 mL centrifuge tube and mixed with 100 µL of the appropriate affinity resin (amylose resin for MBP-GhHA2 and MBP-GhHA2^C329S^ or Ni-NTA agarose for His-KAT1, both pre-equilibrated with lysis buffer) and Triton X-100 at a final concentration of 0.5%. The mixture was incubated on a rotator at 4 °C for 3 h. After incubation, the resin was collected by centrifugation at 4 °C and 4000 rpm for 2 min, and the supernatant was discarded. The resin was washed three times with lysis buffer, each time followed by centrifugation under the same conditions. The resin was then resuspended in an appropriate volume of elution buffer and incubated at 4 °C for 3 h with gentle rotation. After centrifugation, the eluted supernatant was transferred to a sterile 1.5 mL microcentrifuge tube for subsequent use.

### 4.4. S-Nitrosylation Site Prediction and In Vitro S-Nitrosylation Assay

Site Prediction: The GPS-SNO online analysis software [62] (http://sno.biocuckoo.org/) was used for predicting S-nitrosylation sites in amino acid sequences, with the threshold set to “High”. The in vitro S-nitrosylation assay was performed with reference to the method of Chen et al. [11], with appropriate modifications: Thirty micrograms of MBP-GhHA2 recombinant protein was placed in a brown centrifuge tube and treated with HEN buffer (250 mM HEPES, pH 7.7; 4 mM EDTA; 0.1 mM neocuproine) containing 200 μmol GSNO or reduced glutathione for 45 min in the dark. Cold acetone was added to the samples to precipitate the proteins. After washing three times with 70% cold acetone, the protein samples were resuspended in 200 μL of blocking buffer (250 mM HEPES, pH 7.7; 4 mM EDTA; 0.1 mM neocuproine, 2.5% SDS, 20 mM S-methyl methanethiosulfonate [MMTS]), vortexed vigorously for 1 min to mix, and the blocking reaction was carried out in the dark at 50 °C for approximately 1 h. The samples were then precipitated with cold acetone and washed three times with 70% cold acetone. The samples were resuspended in HENS buffer (250 mM HEPES, pH 7.7; 4 mM EDTA; 0.1 mM neocuproine, 1% SDS) and incubated with 50 mM sodium ascorbate (or HENS buffer as a negative control) and 400 μM biotin-HPDP in the dark for 60 min. A portion of the samples was separated by SDS-PAGE, and immunoblotting analysis was performed using an anti-biotin antibody (Cell Signaling Technology, Danvers, MA, USA, Cat No. 7075).

### 4.5. Plasmid Construction and Transformation

The DNA sequence of the silencing interval of *GhHA2* and the CDS coding sequences of *GhHA2* and *GhKAT1* were amplified by PCR using cotton cDNA as a template. The obtained DNA sequence of the silencing interval of *GhHA2* was cloned into the EcoRI and KpnI sites of pYL156 to construct the pYL156-*GhHA2* plasmid. The obtained partial DNA fragment of *GhHA2* was cloned into the EcoRI and SalI sites of pMAL-C2X to construct the pMAL-*GhHA2* plasmid. The partial DNA fragment of *GhKAT1* was cloned into the EcoRI and XhoI sites of pET28a to construct the pET28a-*GhKAT1* plasmid. Using PCR-based site-directed mutagenesis, the pMAL-*GhHA2^C329S^* plasmid was constructed. The recombinant plasmids pMAL-*GhHA2*, pET28a-*GhKAT1*, and pMAL-*GhHA2^C329S^* were transformed into BL21 for prokaryotic expression. The recombinant plasmid pYL156-*GhHA2* was transformed into GV3101 for virus-induced gene silencing (VIGS).

### 4.6. Virus-Induced Gene Silencing (VIGS)

The cotton VIGS experiment was conducted as previously described [63]. The pTRV-RNA1 and pTRV-RNA2 plasmids (pYL156-*GFP*, pYL156-*GhCLA1*, pYL156-*GhHA2*) were introduced into the Agrobacterium tumefaciens strain GV3101. The GV3101 cultures containing the target plasmids were expanded, centrifuged, and resuspended in a buffer (10 mM MES, pH = 5.7; 10 mM MgCl_2_; 200 μM AS) to an OD of 1.5. The resuspended Agrobacterium cultures carrying specific pTRV-RNA1 and pTRV-RNA2 plasmids were mixed in equal volumes and used to infect the cotyledons of 7-day-old cotton seedlings to achieve silencing of the target genes. VIGS-*GhCLA1* plants were used as a visual marker to monitor the silencing efficiency. Samples were taken from the second true leaves of 2-week-old cotton plants for real-time fluorescence quantitative PCR analysis to evaluate the gene-silencing efficiency.

### 4.7. In Vitro Cell-Free Protein Degradation Assay

The in vitro cell-free protein degradation assay was performed with reference to the method of Feng et al. [64] with appropriate modifications. Total leaf proteins were extracted from the leaves of plants treated with water or 500 μM GSNO. The proteins were fully vortexed in a buffer (25 mM Tris, pH = 7.5; 10 mM MgCl_2_; 5 mM DTT; 10 mM NaCl; 10 mM ATP; 0.1% Triton X-100) and centrifuged at 12,000 *g* at 4 °C for 10 min. The supernatant was retained, and the process was repeated twice. Equal amounts of the recombinant protein MBP-GhHA2 and the extracted total proteins were incubated at room temperature. At 0, 15, 30, and 60 min, 20 μL of the mixture was taken, and the protein abundance was analyzed by SDS-PAGE immunoblotting. A treatment with 50 μM MG132 (MCE, Monmouth Junction, NJ, USA) was set up as a control.

### 4.8. Stomatal Aperture Measurement

Measurement of in vivo stomatal aperture in cotton under NO treatment: Under normal water supply conditions, the stomata on the abaxial surface of the second true leaves of cotton plants treated with water or the NO donor 500 μM SNP were isolated using the nail polish imprint method. The abaxial epidermal tissues from the same position on different plants were collected. The air-dried imprints of quick-drying top-coat nail polish (manufactured by Zhejiang Ruili Technology Co., Ltd., Hangzhou, China) were gently placed on a glass slide, covered with a coverslip, and gently pressed with the thumb. Images were taken under a 40× objective lens, and the stomatal aperture was measured.

Measurement of stomatal aperture in excised leaves of VIGS-silenced plants under NO treatment: The second true leaves of cotton plants under different treatments were taken, and the abaxial epidermal tissues from the same position on different plants were isolated as much as possible and soaked in MES-KOH buffer (10 mM MES, 50 mM KCl, 10 mM CaCl_2_, pH 6.15) for 3 h in a light incubator to ensure full opening of the stomata. Then, 200 μM SNP was added, and the leaves were treated in the light incubator for 3 h. The chloroplasts on the epidermal tissues were brushed off with a paintbrush (to eliminate the interference of leaf background pigments and cell impurities, effectively reducing visual obstruction), placed on a glass slide, covered with a coverslip, gently pressed with the thumb while absorbing excess water with absorbent paper, and images were taken under a 20× objective lens to record the stomatal aperture of different materials under different treatments. The operation process was carried out as quickly as possible. The stomatal aperture was measured using Image J (1.54d) software. All experiments were performed with three independent biological replicates (*n* = 3), with at least 60 stomata quantified. The data are shown as means ± SD.

### 4.9. RNA Extraction and Real-Time Fluorescence Quantitative RT-qPCR for Gene Expression Analysis

Detection of gene silencing efficiency: The second true leaves of cotton plants approximately 2 weeks after VIGS (when the albino phenotype appeared) were taken. RNA was extracted from the leaf tissues of both control and silenced plants using the Beijing Tsingke RNA extraction kit, and cDNA was synthesized by reverse transcription. The gene silencing efficiency was analyzed by real-time fluorescence quantitative PCR. The PCR program was as follows: denaturation at 94 °C for 30 s; 40 cycles of denaturation at 94 °C for 5 s; and annealing at 60 °C for 34 s. The cotton GhActin9 gene was used as a control. The relative expression level was calculated using the 2^−ΔΔCt^ method.

Analysis of endogenous antioxidant enzyme genes in cotton: The antioxidant enzyme genes in cotton were *GhAPX (Gh_A08G1746)*, *GhGPX (Gh_A07G0269)*, *GhCAT (Gh_D03G0021)*, and *GhPOD (Gh_A08G0714)*. When the cotton plants reached the two-leaf stage, the second true leaves at different time points after treatment with the NO donor SNP were collected and immediately frozen in liquid nitrogen. Total RNA was extracted using the TSINGKE RNA extraction kit, and the RNA was reverse transcribed into cDNA according to the instructions of the TaKaRa (Kusatsu, Japan) reverse transcription kit. The expression level of the target genes was corrected using *GhActin9* as an internal reference gene, and the relative expression level was calculated using the 2^−ΔΔCt^ method. All experimental treatments were set up with three biological replicates. All experiments were performed with three independent biological replicates (*n* = 3). The data are shown as means ± SD.

### 4.10. Measurement of Relative Water Content

The second true leaves of cotton plants under different treatments were taken, and the fresh weight (FW) of each leaf was weighed. Then, the leaves were soaked in deionized water for 24 h, and the excess water was absorbed with absorbent paper. The saturated weight (TW) was weighed and recorded. The weighed leaves were dried to a constant weight, and the dry weight (DW) was weighed and recorded. The relative water content of the leaves was calculated as follows: RWC (%) = [(FW − DW)/(TW − DW)] × 100. Four biological replicates were set up for the experiment. All experiments were performed with four independent biological replicates (*n* = 4). The data are shown as means ± SD.

### 4.11. Determination of Water Loss Rate

The aboveground parts of cotton plants under different treatment conditions were taken, and the initial fresh weight (W_0_) of the aboveground parts was weighed and recorded. Then, the plants were placed on a table with uniform light and good ventilation. The fresh weight (W_n_) of the aboveground parts of the plants was recorded at different time points. The water loss rate (%) = [(W_0_ − W_n_)/W_0_] × 100. Three biological replicates were set up for the experiment. All experiments were performed with three independent biological replicates (*n* = 3). The data are shown as means ± SD.

### 4.12. Measurement of Leaf Photosynthetic Rate and Calculation of Water-Use Efficiency

The photosynthetic rate was measured using a LI-6400 portable photosynthesis system (LI-COR, Lincoln, NE, USA). The leaves were placed in the leaf chamber, and the data were recorded after the values stabilized. The measurement parameters were set as follows: a “2 × 3 LED” red–blue light source, a leaf chamber area of 6 cm^2^, an air flow rate of 500 μmol/s, a temperature of 25 °C, and a light intensity of 200 μmol·m^−2^·s^−1^. All experiments were performed with four independent biological replicates (*n* = 4). The data are shown as means ± SD.

### 4.13. Infrared Thermal Imaging Analysis

Cotton plants under normal water supply conditions were sprayed with water or the NO donor SNP. After 3 h, thermal imaging images were captured using an infrared thermal imager (VarioCAM HD, InfraTec GmbH, Dresden, Germany), and the images were analyzed and processed using the InfraTec IRBIS 3 software (http://www.infratec.cn).

### 4.14. Measurement of Leaf Water Potential

The experiment was conducted as previously described by He et al. [65]. The second true leaves of cotton plants under different stress conditions were taken and immediately placed in a pressure chamber (Model 3000, Soil Moisture Equipment Corp., Goleta, CA, USA). Pressure was gradually applied until a droplet appeared at the petiole. The pressure was immediately stopped, and the negative value of the pressure gauge reading at this time was recorded as the leaf water potential. All experiments were performed with four independent biological replicates (*n* = 4). The data are shown as means ± SD.

### 4.15. Fluorescence Staining and Confocal Microscopic Imaging Analysis

Leaf discs of cotton plants under different treatments were collected and placed in 25 μM PI staining solution or 25 μM H_2_DCFDA staining solution (containing 140 mM NaCl, 2.7 mM KCl, 10 mM Na_2_HPO_4_, 1.8 mM KH_2_PO_4_) and incubated at 37 °C for 30 min. After washing with 1× PBS (pH = 7.4), the samples were imaged using an LSM 900 laser confocal microscope under excitation light channels of 488 nm or 561 nm. The relative fluorescence signal intensity was quantitatively analyzed using Image J (1.54d) software to quantify the fluorescence intensity in the confocal images. One-way analysis of variance was used for quantitative evaluation, and different letters indicate significant differences at the *p* < 0.05 level.

### 4.16. Exogenous Spraying and Drought Treatment

For exogenous spraying treatment, 500 μM Sodium Nitroprusside (III) Dihydrate (SNP) was uniformly sprayed on both the adaxial and abaxial surfaces of the leaves of two-leaf-stage seedlings, and the control group was sprayed with an equal amount of deionized water. For drought stress treatment, watering of normally growing cotton plants in the greenhouse was stopped for 1 week, and the plants were photographed when the cotton leaves showed wilting symptoms.

### 4.17. Statistical Analysis

The data were processed using Microsoft Excel 2016, and one-way analysis of variance or *t*-tests were performed on the experimental data using IBM SPSS 22.0 software. Significance was set at *p* < 0.05. Duncan’s method was used for multiple comparisons of means, and significant differences are indicated by different lowercase letters.

## 5. Conclusions

This study confirms that exogenous NO significantly enhances cotton drought tolerance through a dual synergistic mechanism: first, NO upregulates the temporal expression of antioxidant enzyme genes such as *GhCAT*, *GhAPX*, *GhPOD*, and *GhGPX*; enhances enzyme activities; efficiently scavenges ROS induced by drought; and alleviates oxidative damage. Second, NO mediates S-nitrosylation modification of plasma membrane H^+^-ATPase GhHA2 at the Cys329 site; inhibits its proteasomal degradation; and enhances protein stability, thereby promoting stomatal closure, reducing transpirational water loss, and maintaining water balance. GhHA2 positively regulates cotton drought tolerance, and its silencing impairs the NO-mediated stomatal closure response, decreasing the photosynthetic rate and water-use efficiency. This study clarifies the synergistic role of NO-mediated antioxidant defense and targeted GhHA2 modification in conferring drought resistance, providing a novel target for enhancing crop drought tolerance through genetic engineering.

## Data Availability

Data will be made available on request.

## Supplementary Materials

The following supporting information can be downloaded at: https://www.mdpi.com/article/10.3390/plants15101463/s1, Figure S1: Effects of exogenous SNP application on leaf water potential in cotton; Figure S2: Relative expression level of *GhHA2* in VIGS-*GhHA2*-silenced cotton plants; Figure S3: Effects of exogenous SNP application on cotton seedlings under PEG-simulated drought stress; Figure S4: In vitro S-nitrosylation assay of GhKAT1 protein; Figure S5: Expression patterns of *GhHA2* and *GhKAT1* genes over time under SNP treatment; Table S1: Key substrate proteins identified by S-nitrosoproteomics in cotton (*Gossypium hirsutum* L.) under SNP treatment; Table S2: List of primers used in this study.

## Abbreviations

The following abbreviations are used in this manuscript:

ROS	Reactive oxygen species
BAK1	BRI1-associated receptor kinase
ABA	Absicsic Acid
CBB	Comassie brilliant blue
PS	Ponceau S
PEG	Polyethylene glycol
MES	2-N-morpholinoe thanesulfonate
CCD	Cold charge-coupled device
PAGE	Polyacrylamide gel electrophoresis
ATP	Adenosine triphosphate
MBP	Maltose-binding protein
MG132	Carbobenzoxy-L-leucyl-L-leucyl-L-leucinal
qRT-PCR	Quantitative real-time PCR
WT	Wild type
cm	centimeter
g	gram
h	hour
min	minute
mL	milliliter
rpm	rounds per minute
