# Supplementary material for: Nitric Oxide Enhances Drought Tolerance in Gossypium hirsutum L. via S-Nitrosylation of the Plasma Membrane H+-ATPase Isoform GhHA2 and Antioxidant Defense Activation
Source: Plants (Basel). 2026 May 11;15(10):1463. doi: 10.3390/plants15101463 (PMC13210433; doi:10.3390/plants15101463)
Supplement: Supplementary file 1 [file plants-15-01463-s001.zip › plants-4255749-supplementary.pdf]

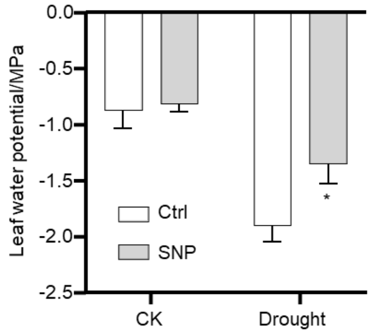

Figure S1 Effects of exogenous SNP application on leaf water potential in cotton

Changes in leaf water potential (MPa) of cotton plants under different treatments. CK, well-watered control; Drought, drought-stressed condition; SNP, exogenous sodium nitroprusside (500 μM, a nitric oxide donor) application under drought stress. Asterisks (*) indicate statistically significant differences between treatments ($p < 0.05$, t-test). Data represent means ± SD of four biological replicates ($n = 4$).

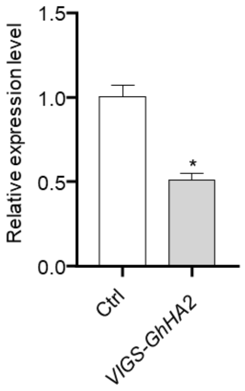

Figure S2 Relative expression level of *GhHA2* in VIGS-*GhHA2* silenced cotton plants

Relative expression levels of *GhHA2* in cotton following virus-induced gene silencing (VIGS). Ctrl (VIGS-*GFP*), negative control expressing green fluorescent protein; VIGS-*GhHA2*, *GhHA2* silencing treatment. Relative expression was quantified by qRT-PCR using *GhActin9* as the internal reference gene. Asterisks (*) indicate statistically significant differences determined by t-test ($p < 0.05$). Data represent means ± SD of three biological replicates ($n = 3$).

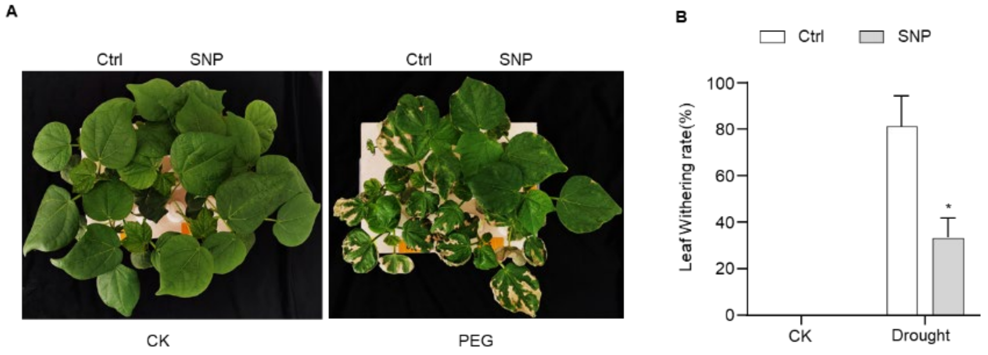

Figure S3 Effects of exogenous SNP application on cotton seedlings under PEG-simulated drought stress

A. Phenotypic analysis of cotton plants under different treatments. CK, well-watered control (hydroponic); PEG, drought stress simulated with 10% polyethylene glycol (PEG-6000); SNP, exogenous sodium nitroprusside (500 μM) application under drought stress. B. Leaf withering rate (%) under

corresponding treatments shown in panel a. Asterisks (*) indicate statistically significant differences between treatments ($p < 0.05$, t-test). Data represent means ± SD of three biological replicates ($n = 3$).

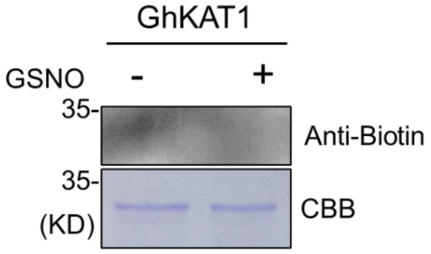

Figure S4 In vitro S-nitrosylation assay of GhKAT1 protein

Detection of S-nitrosylated GhKAT1 by biotin-switch assay after GSNO treatment. S-nitrosylated proteins were labeled using the biotin-switch method and detected by immunoblotting with anti-biotin antibody. Coomassie brilliant blue (CBB) staining of the membrane indicates equal protein loading.

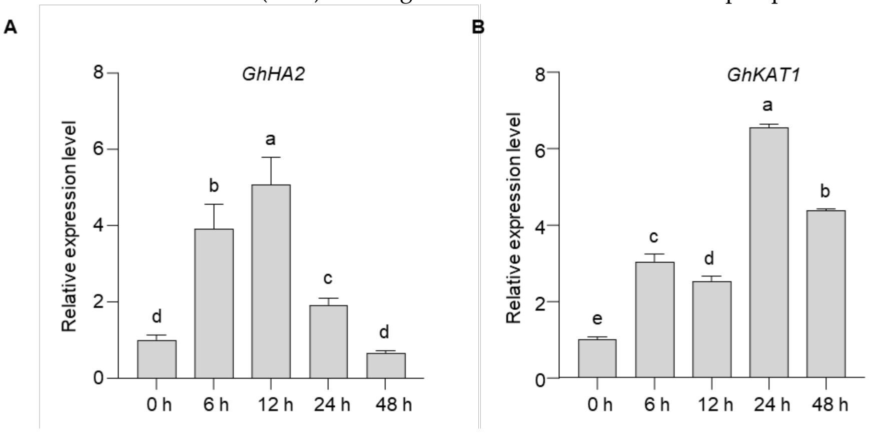

Figure S5 Expression patterns of *GhHA2* and *GhKAT1* genes over time under SNP treatment

(A, B) Relative expression levels of GhHA2 (A) and GhKAT1 (B) measured at 0 h, 6 h, 12 h, 24 h, and 48 h after treatment. The relative expression levels were normalized to the expression at 0 h. Data are presented as the mean ± SD of three independent biological replicates ($n = 3$). Different lowercase letters indicate significant differences among different time points at $p < 0.05$, as determined by one-way ANOVA followed by Duncan's multiple range test.

Table S1 Key substrate proteins identified by S-nitrosoproteomics in cotton (*Gossypium hirsutum L.*) under SNP treatment.

Protein accession	Position	Amino acid	Protein description
Gh_A07G0269	117	C	Glutathione peroxidase OS=*Gossypium hirsutum* OX=3635 GN=LOC107953728 PE=3 SV=1
Gh_D03G0021	232	C	Catalase OS=*Gossypium hirsutum* OX=3635 GN=LOC107937827 PE=3 SV=1
Gh_A08G0714	200	C	Peroxidase OS=*Gossypium hirsutum* OX=3635 GN=LOC107925084 PE=3 SV=1
Gh_A08G1746	46	C	L-ascorbate peroxidase OS=*Gossypium hirsutum* OX=3635 GN=LOC107942909 PE=3 SV=1
Gh_D03G0021	88	C	Catalase OS=*Gossypium hirsutum* OX=3635 GN=LOC107937827 PE=3 SV=1
Gh_A08G0714	68	C	Peroxidase OS=*Gossypium hirsutum* OX=3635 GN=LOC107925084 PE=3 SV=1

List of key substrate proteins identified via S-nitrosoproteomics in *Gossypium hirsutum* under SNP treatment. The table details the corresponding gene ID, the position of the S-nitrosylated cysteine residue, the amino acid residue, and the functional annotation for each protein.

Table S2 List of primers used in this study.

Cloning primer	F (5′-3′)	R (5′-3′)
pMAL-*GhHA2*	gagggaaggatttcagaattcGGGTCTCACA GGCTGTCTCAGC	caagcttgcctgcaggtcgacGTTCTTC ATCCTTTGGAAAATGGC
pMAL-*GhHA2*^{C329S}	GATGTCCTTaGCAGTGATAAGACA GGAACATTGAC	TCACTGCtAAGGACATCCAT ACCGGCCATTTC
pET28a-*GhKAT1*	atgggtcgcggatccgaattcATGTGTAGCC TTGAGTATTCAAACAAC	gtggtggtggtggtgctcgagCTCCCA TGCCCTGTAGTAAGGG
pYL156-*GhHA2*	gtgagtaaggttaccgaattcACTGTCATATT CTTCTGGGCCATG	gagacgcgtgagctcggtaccTATGAC ACCAGCCCATCCCC
**RT-qPCR**	**F (5′-3′)**	**R (5′-3′)**
qRT*GhHA2*	TCAGACGAGGGAGTCAACAG	CGACGAAATCTTGCCAATC
Gh*Actin9*	GCCTTGGACTATGAGCAGGA	AAGAGATGGCTGGAAGAGG A
qRT*GhAPX*	TCCTATCCTTTCATACGCTGAC	TAGCATTGGGAAGACGACC
qRT*GhCAT*	TGACATCGGTGTTCCACAG	CTTATGGCTTCATCCTCCAA C
qRT*GhGPX*	ATGTGAATGGACCGAATACG	ATGTCGTTGGTGGATACCTC T
qRT*GhPOD*	TGTTCAAGGATGTGATGCG	AAGCACTCTCTAAGGCAGC C
qRT*GhKAT1*	TGGATGCTGAGTATTTCCCAC	TGCTACCTTTCCCATAACCT G
qRT*GhHA2*	TCAGACGAGGGAGTCAACAG	CGACGAAATCTTGCCAATC
